# Janus Hydrogel Microbeads for Glucose Sensing with pH Calibration

**DOI:** 10.3390/s21144829

**Published:** 2021-07-15

**Authors:** Maru Ando, Mio Tsuchiya, Shun Itai, Tomomi Murayama, Yuta Kurashina, Yun Jung Heo, Hiroaki Onoe

**Affiliations:** 1 Westminster School, London SW1P 3PB, UK; amaru0304@gmail.com; 2 Department of Mechanical Engineering, Faculty of Science and Technology, Keio University, Kanagawa 223-8522, Japan; mio19951011@a8.keio.jp (M.T.); shun.i@keio.jp (S.I.); t.murayama@keio.jp (T.M.); kurashina.y.aa@m.titech.ac.jp (Y.K.); 3 Department of Materials Science and Engineering, School of Materials and Chemical Technology, Tokyo Institute of Technology, Yokohama 226-8503, Japan; 4 Department of Mechanical Engineering, Collage of Engineering, Kyung Hee University, Yongin 17104, Korea; yunjheo@khu.ac.kr; 5 Integrated Education Institute for Frontier Science & Technology (BK21 Four), Kyung Hee University, Yongin 17104, Korea

**Keywords:** glucose sensor, glucose monitoring, pH calibration, hydrogel, Janus particle

## Abstract

We present fluorescent Janus hydrogel microbeads for continuous glucose sensing with pH calibration. The Janus hydrogel microbeads, that consist of fluorescent glucose and pH sensors, were fabricated with a UV-assisted centrifugal microfluidic device. The microbead can calibrate the pH values of its surroundings and enables accurate measurements of glucose within various pH conditions. As a proof of concept, we succeeded in obtaining the accurate value of glucose concentration in a body-fluid-like sample solution. We believe that our fluorescent microbeads, with pH calibration capability, could be applied to fully implantable sensors for continuous glucose monitoring.

## 1. Introduction

Continuous glucose monitoring (CGM) has been gaining attention, since it enables people with diabetes to constantly monitor blood glucose concentration [1]. The use of CGM and the identification of glucose trends have been expected to help build suitable treatment plans and to assist clinicians in case of emergency. Most commercial CGM devices are semi-implantable [2,3]. They need to inject sensing needles under the skin and attach a needle-connected transmitter onto the skin [4]. Although these CGM devices can measure glucose concentration accurately, the open wound and the wearing of the device in daily life can sometimes increase the risk of potential infection [5] and the burden for the patients [6]. In addition, the wired connection of semi-implantable devices is not favorable for patients’ physical activities [7].

To overcome these issues, one promising method is a fully implantable glucose sensor. Among the various fully implanted sensor systems, fluorescent microbeads [8] provide minimally invasive glucose monitoring. Glucose-responsive fluorescence immobilized in microbeads reversely responds to glucose concentration, changing the fluorescence intensity depending on the glucose concentration. Boronic acid-based glucose-responsive fluorescence is stable for up to 140 days in vivo [9]. Furthermore, the fluorescent signal can be measured through the skin. These features are attractive for reducing the burdens of patients. However, the surrounding pH value of the implanted sensors can interfere with the intensity of fluorescence, leading to inaccuracies in the readings of glucose concentration in the body, where the pH value is not always constant; physiological pH increases due to inflammation and decreases due to exercise [10,11,12]. Especially after implantation, in vivo environment, including acute inflammation, hinders the stable response of a CGM device. Thus, the simultaneous sensing of glucose and pH is required for implantable sensors to maintain accuracy even when pH changes at an implantation site.

**Figure 1:**
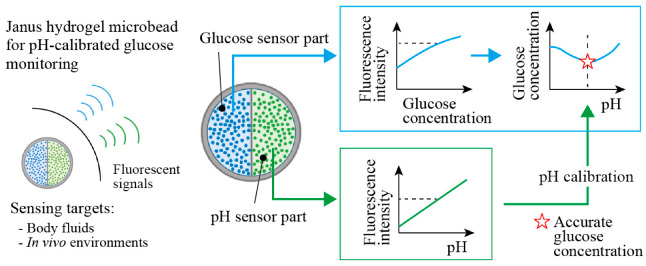
Conceptual illustration of Janus hydrogel microbeads for sensing glucose concentration with pH calibration. The microbeads are composed of fluorescent glucose sensor and pH sensor in each hemisphere. The fluorescence intensity of the glucose sensor part provides accurate glucose concentration readings, with calibration by the surrounding pH obtained from the fluorescence intensity of the pH sensor part.

Here, we propose Janus fluorescent microbeads that encapsulate two fluorescent moieties, which are responsive to glucose and pH in two different compartments (Figure 1). These functional moieties change their fluorescence intensities with the glucose concentration and the pH values; thus, more accurate glucose concentration measurements are enabled by measuring both fluorescence intensities, with the calibration using the pH value (Figure 1 right). We use a centrifuge-based droplet shooting device [13,14,15] for fabricating Janus fluorescent microbeads easily and rapidly. The sensing performance of our fabricated Janus microbeads is evaluated to demonstrate the ability to measure glucose concentration in a body-fluid-like sample solution with pH calibration.

## 2. Materials and Methods

### 2.1. Chemicals

Sodium alginate (194-13321), calcium chloride (090-00475), glucose (049-31165), acrylamide (016-00765) and mehylenebisacrylamide (134-02352) were purchased from Wako Pure Chemical Industries (Osaka, Japan). Glucose-responsive monomer was presented by LG Chem Ltd. pH-responsive monomer, Fluorescein-5-Thiosemicarbazide (F-121), was purchased from Thermo Fisher Scientific. Disodium hydrogen phosphate (U0858) and sodium dihydrogen phosphate (U1886) were purchased from Sigma Aldrich. Photoinitiator (Irgacure 1173) was purchased from BASF. Ethylenediaminetetraacetic acid (EDTA) (341-01622) was purchased from Dojindo. Deionized (DI) water was purified with the Millipore Purification System.

### 2.2. Centrifuge-Based Fabrication of Hydrogel Microbeads

The multifunctional Janus hydrogel microbeads were fabricated by the ultraviolet (UV)-light-assisted centrifugal droplet shooting method [15] with one step. The centrifuge-based droplet shooting part (CDSD) was composed of a theta capillary (G-1.5, Narishige), a lab-made capillary-holder and a 1.5 mL microtube. The theta capillary was pulled using a puller (PC-10, Narishige) and cut using a microforge (MF-900, Narishige) to obtain a capillary orifice with a desired diameter (80–100 µm). The ultraviolet light-emitting diode (UV-LED) part for centrifuging was composed of a 3D-printed jig with UV-LEDs (UF3VL-1H411, DOWA) and electric wirings to supply electric current to the UV-LEDs. The jig with four UV-LEDs was inserted into a 50 mL centrifuge tube (VIO-15BN, VIOLAMO). Electric power was supplied to the UV-LEDs via a slip ring (EC4294-2, Moog Components Group) that was attached to a centrifuge (H-19R, Kokusan).

Pre-gel solution for a glucose-sensing hemisphere was prepared by mixing 15% acrylamide, 0.3% methylene bisacrylamide, 2.5% sodium alginate, 5% glucose-responsive monomer and 0.5% photoinitiator. Pre-gel solution for a pH-sensing hemisphere was prepared by mixing 20% acrylamide, 0.3% methylene bisacrylamide, 2.5% sodium alginate, 0.5% pH-responsive monomer [16] and 0.5% photoinitiator. These pre-gel solutions were separately introduced into each segment of the pulled theta capillary. Then, the theta capillary was assembled with the lab-made capillary holder that was inserted into the microtube filled with 0.15 mol/L CaCl_2_ solution. The pre-gel solution in the theta capillary was ejected by centrifugation at 160 G for 60 seconds while irradiating UV, to fabricate hydrogel microbeads with a Janus structure.

### 2.3. Observation and Evaluation of Hydrogel Microbeads

The fabricated microbeads were observed by using an inverted phase-contrast/fluorescent microscope (IX73P1-22FL/PH, Olympus, Tokyo, Japan) and a confocal laser scanning microscope (FV3000, Olympus, Tokyo, Japan). The obtained phase-contrast images were analyzed using ImageJ (National Institutes of Health, Bethesda, MD, USA) to obtain the diameter values of the microbeads for analyzing the diameter distribution.

### 2.4. Glucose and pH Response Experiments

We firstly prepared two buffer solutions, 0.06 mol/L Na_2_HPO_4_ and 0.06 mol/L NaH_2_PO_4_, with 0.001 mol/L EDTA. By mixing these two buffer solutions, we made five different solutions with pH values from 4.0 to 8.0. Glucose was then added to these solutions to obtain the final solutions with different pH values and glucose concentrations.

For the characterization of the glucose-sensing part of the microbeads at a neutral pH condition, glucose was added to the buffer solutions at pH 7.0 to prepare 0, 2, 5, 10, 20, 40, 80, 160, 320 and 640 mg/dL glucose concentrations. For the evaluation with various pH conditions, the fluorescence intensities of the microbeads were measured in buffer solutions with different glucose concentrations (0, 50, 100, 200, 300, 400 and 500 mg/dL) and pH values (pH 4.0, 5.0, 6.0, 7.0 and 8.0). 

The fabricated Janus hydrogel microbeads were washed with DI water several times, rinsed with sample solution and then immersed for 10 min. After that, the microbeads were observed using the inverted fluorescent microscope and the confocal laser scanning microscope (FV3000, Olympus, Tokyo, Japan). The obtained images were analyzed using ImageJ to measure the fluorescence intensities. In the case of the characterization of the glucose-sensing parts, the measured fluorescence intensities were normalized from 0 to 1 by the maximum fluorescence intensity. The mean values of the fluorescence intensities of the glucose-sensing hemispheres and the pH-sensing hemisphere of the microbeads in the prepared buffer solution were plotted on three-dimensional graphs.

### 2.5. Demonstration of Glucose Measurement with pH Calibration

As a body-fluid-like solution, we prepared three different pH-adjusted sample solutions of a diluted beverage (POCARI SWEAT^®^, Otsuka Pharmaceutical) with DI water supplemented with 0.001 mol/L EDTA. The fabricated Janus fluorescent microbeads were washed with DI water several times, rinsed with the sample solutions and then immersed in the sample solution for 10 min and observed using the inverted fluorescent microscope. The obtained images were analyzed using ImageJ (National Institutes of Health, Bethesda, MD, USA) to measure fluorescence intensity of a glucose-sensing hemisphere and a pH-sensing hemisphere.

## 3. Results

The proposed Janus fluorescent hydrogel microbeads encapsulate two different fluorescent moieties responsive to glucose and pH in each hemispherical compartment to enable accurate continuous glucose monitoring, minimizing the surrounding pH interference (Figure 1). The pH value obtained from the pH-sensing hemisphere can be used for calibrating the obtained glucose concentration with the fluorescence intensity of the glucose-sensing hemisphere. 

**Figure 2:**
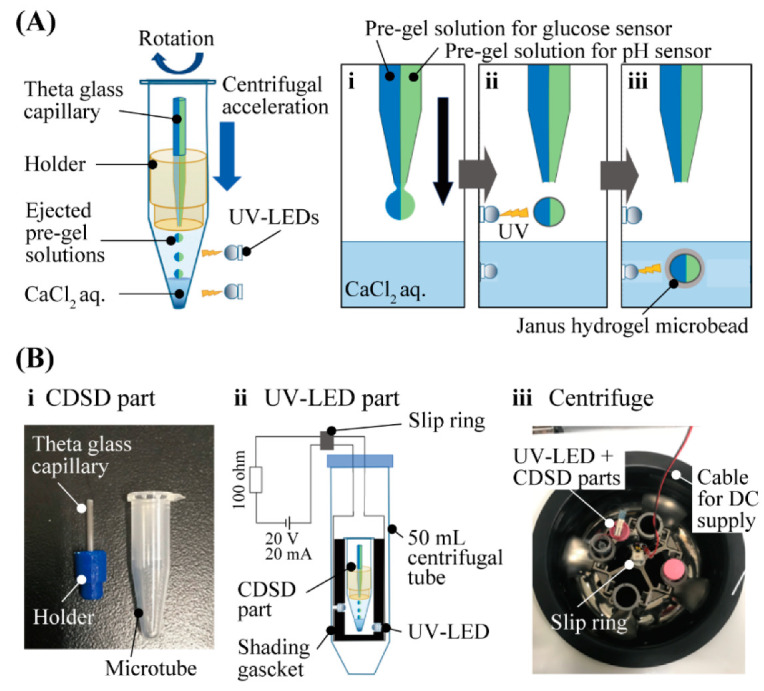
Fabrication of Janus hydrogel microbead sensor with CDSD with UV irradiation (**A**) Fabrication principle of Janus hydrogel microbeads. (**B**) Setup for the fabrication process.

The principle and method of fabricating Janus hydrogel microbeads (Figure 2A) is based on the previously reported method involving CDSD with UV-LED [15]. We introduced two different pre-gel solutions into each barrel of a theta capillary. The pre-gel solution for the glucose-sensing hemisphere was the mixture of glucose-responsive fluorophore, acrylamide and sodium alginate with crosslinker and photoinitiator. For the pH-sensing hemisphere, the pH-responsive fluorophore, Fluorescein-5-thiosemicarbazide, was used instead of the glucose-responsive fluorophore. Both the pre-gel solutions were ejected from the tip of the theta capillary by a centrifugal force to form micro-droplets of the pre-gel solutions. Just after the ejection, the micro-droplets were irradiated by UV light to proceed to the photopolymerization of the acrylamide and fluorophores in the air. Immediately after the micro-droplets dove into the CaCl_2_ solution at the bottom of the microtube, alginate was ionically cross-linked in the CaCl_2_ solution to form Janus hydrogel microbeads. The setup for the microbead fabrication composed of a CDSD part and a UV-LED part (Figure 2B). The CDSD part (Figure 2B-i) including the theta capillary (tip diameter: 80–100 µm) fixed to a 1.5 mL microtube with the holder was set in the UV-LED part (Figure 2B-ii). An electric current for UV light irradiation was sent from a DC power supply to the four LEDs (0.9 mW) through a slip ring during centrifugation (Figure 2B-iii).

**Figure 3:**
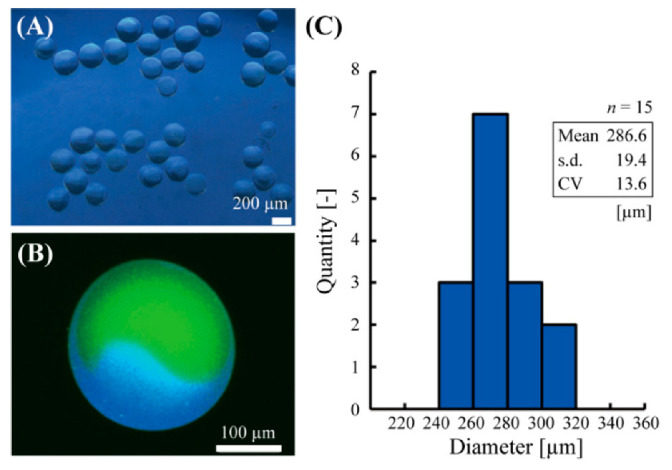
Fabricated Janus hydrogel microbeads. (**A**) A phase-contrast microscopic image of the microbeads. (**B**) A confocal fluorescent microscopic image of the microbeads. (**C**) Diameter distribution of the fabricated microbeads.

Using this method, the Janus hydrogel microbeads with glucose sensor and pH sensor were fabricated in a single centrifugal step (Figure 3A). Confocal fluorescent microscopic observation confirmed the Janus structure of the microbeads with the pH sensor part (green) on one side and glucose sensor part (blue) on the other side (Figure 3B). A thin alginate hydrogel shell, whose thickness could be controlled with UV irradiation power or duration, was also observed, similarly to the previous literature [15]. The diameter of the microbeads was 286.6 ± 19.4 μm (mean ± s.d.) with a CV of 6.8% (Figure 3C). 

**Figure 4:**
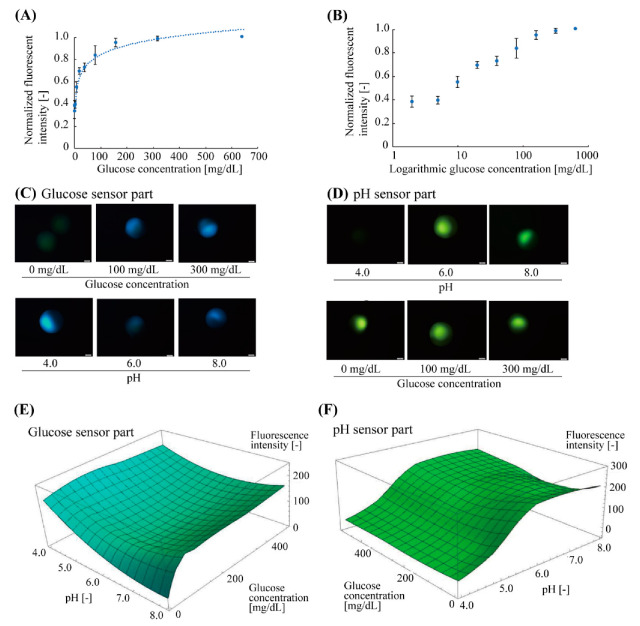
Fluorescent characteristics of glucose and pH sensor parts of the microbeads. (**A**) Relationship between normalized fluorescence intensity and glucose concentration of the glucose sensor parts at neutral pH (pH 7.0). The plots and error bars indicate the mean and standard deviation, respectively (*n* = 4). The dotted curve is a logarithmic correlation curve. (**B**) Semi-logarithmic plot of (**A**). The approximate measurable range of the glucose concentration with the microbead sensor were 10–320 mg/dL. (**C**) Fluorescent images of glucose sensor part in various glucose concentrations (0–300 mg/dL, pH 7) and pH conditions (pH 4.0–8.0, 200 mg/dL glucose) (**D**) Fluorescent images of pH sensor part in various pH conditions (pH 4.0–8.0, 500 mg/dL glucose) and glucose concentrations (0–300 mg/dL, pH 6). (**E**,**F**) Fluorescent characteristics of the glucose and pH sensor parts for varied pH and glucose concentrations.

Before glucose measurement with pH calibration, we confirmed the fluorescent characteristics of the glucose-sensing part at a neutral pH condition (pH = 7.0) (number of microbeads, *n* = 4) (Figure 4A,B). The glucose-sensing part exhibited a non-linear response to the glucose concentration ranging from 2 mg/dL to 640 mg/dL. The correlation curve fitted to the plots was a logarithmic function, *y* = 0.12 log *x* + 0.27, (*R*^2^ = 0.96), indicated by the dotted line (Figure 4A). The standard deviation of each plot (averaged value) was less than 10%. The approximate range of the measurable glucose concentration was estimated to be 10–320 mg/dL (Figure 4B).

Next, the fluorescent response of the fabricated microbead to pH and glucose concentration was evaluated (Figure 4C–F). The Janus hydrogel microbeads were immersed in the buffer solutions with various pH (pH = 4.0–8.0) and glucose concentrations (0–500 mg/dL). The fluorescence intensities of the glucose-responsive part (blue fluorescence: *λ*_em-peak_ = 488 nm) and pH-responsive part (green fluorescence: *λ*_em-peak_ = 521 nm) were measured separately. These fluorescent measurements revealed that the fluorescence intensity of the glucose sensor part increased with the increase in glucose concentration (Figure 4C top), but the intensity was interfered with by the pH value even at the same glucose concentration: the fluorescence intensity became higher at low and high pH regions (pH 4.0 and 8.0) than it did around the neural pH region (pH 6.0) (Figure 4C bottom). This result indicates that it could be difficult to obtain the accurate concentration with only the fluorescence intensity. On the other hand, the fluorescence intensity of the pH sensor part also increased with the increase in the pH values (Figure 4D top) and kept almost constant to the changes in the glucose concentration (Figure 4D bottom) (less than 11% intensity fluctuation to 0–500 mg/dL glucose). These fluorescent characteristics of the glucose-sensing parts and the pH-sensing parts were plotted to obtain three-dimensional (3D) graphs (Figure 4E,F). Using these graphs, the glucose concentration that is calibrated with the interference due to the surrounding pH can be obtained by the measured fluorescence intensities of both of the sensor parts.

**Figure 5:**
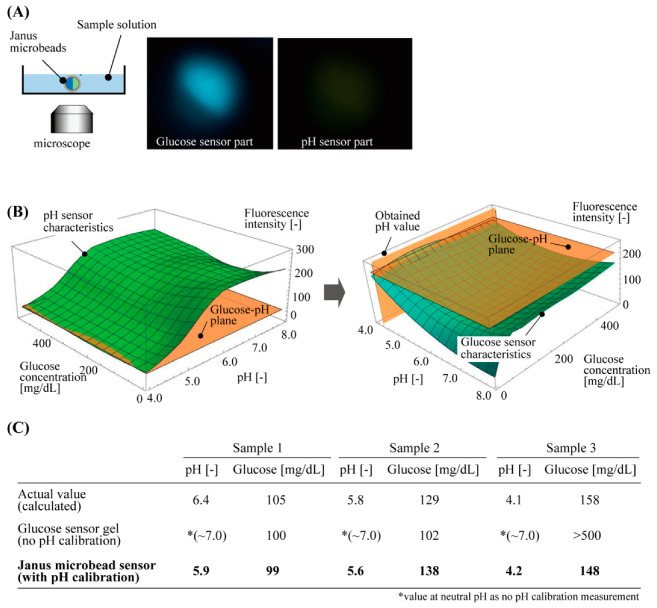
Demonstration of glucose concentration measurement of sample solutions by using Janus hydrogel microbeads with pH calibration. (**A**) Experimental setup and typical obtained fluorescent images of the microbeads. (**B**) Procedures for obtaining glucose concentration with the fluorescent characteristics of the Janus hydrogel microbeads. (**C**) Comparison of the values of glucose concentration obtained by actual content calculation, glucose sensor gel without pH calibration, and Janus microbead sensor with pH calibration.

To demonstrate the glucose measurement with pH calibration, we applied our sensor to three different sample aqueous solutions of a diluted beverage, which included various salt components, similar to our body fluids, as well as glucose. The fabricated fluorescent microbeads were immersed in the sample solutions in each dish. The intensities of fluorescence were measured for the glucose-responsive part and pH-responsive part simultaneously by using the fluorescent microscope (Figure 5A).

These measured fluorescence intensities were processed with the two 3D graphs above: one for the pH-responsive part and the other for the glucose-responsive part (Figure 5B). The steps to obtain the calibrated glucose concentration were as follows: the 3D graph for the pH-responsive part was cut at the plane of the measured fluorescence intensity of the pH sensor part, to obtain the pH value of the solution from the glucose–pH plane (Figure 5B left). Using this pH value, the other 3D graph for the glucose-responsive part was cut at the plane of the measured fluorescence intensity of the glucose sensor part and the pH value to obtain the glucose concentration of the solution (Figure 5B right). These measured values of the pH and glucose had less than 0.5 difference in pH and less than 10 mg/dL difference (<10% error) in glucose concentration, as compared to the calculated actual glucose concentration of the sample solution. However, a large difference was measured for the values of glucose concentration that were only estimated with the fluorescence intensity of the glucose sensor part without pH calibration (the value of the fluorescence intensity at neutral pH, 7.0) (Figure 5C). This result indicates that the accuracy of the glucose concentration measurement was improved by the new fluorescent microbeads with a pH calibration function.

## 4. Discussion

In this study, we proposed a Janus microbead fluorescent glucose sensor with pH calibration. The present microbeads have sufficient sensitivity for clinical use. Boronic acids have a low LOD (limit of detection) (>0.2 mg/dL) [17,18]. The required glucose sensor accuracy (15% MARD (mean absolute relative difference) in <70 mg/dL) is much higher than the LOD value because double-digit mg/dL detection levels are sufficient for insulin bolus calculation. We evaluated the sensing performance of our fabricated Janus microbeads in a body-fluid-like solution and demonstrated their capability to measure glucose concentration within <10% error in the range of 100–160 mg/dL (Figure 5C), which is a typical glucose concentration range of interest in blood (fasting: <110 mg/dL, 75 g OGTT (oral glucose tolerance test): <140 mg/dL). Previously, multi-sensing Janus particles were developed for the detection of glucose and H_2_O_2_ [19], but the purpose of this previous work was the simultaneous detection of two different substances in order to display the glucose concentration as a visible color. In the present study, our particles can use the pH value obtained from one compartment for the calibration of a glucose sensor contained in the other compartment, which the previously proposed glucose sensor microbeads [8,15,19] have not achieved. Therefore, this unique Janus fluorescent microbead with pH calibration can provide highly accurate CGM even during exercise or periods of inflammation. For practical applications, the sensor’s toxicity and long-term stability in vivo should be considered. A previous report shows that a modification using poly-ethylene glycol (PEG) for the glucose-sensing fluorescent hydrogel reduced the body immune reaction and increased the sensor stability in vivo over 140 days [9]. This PEG modification could also be applicable and effective for our Janus microbead sensor for in vivo uses.

The fluorescence intensity of the boronic acid-based glucose-sensing hydrogel shows a non-linear response to the glucose concentration, as shown in Figure 4A,B,E. Thus, our sensor needs the characterization of the fluorescence intensity to the glucose concentration, which determines the accuracy of the glucose concentration measurement. In addition, as the pH decreased to less than 5.0, the fluorescent response of the glucose-sensing part became gradually lower, causing difficulty in keeping the required dynamic range of the glucose measurement for diabetes. Although most of the pH changes in our body caused by exercise are above the pH of 6.5 [10], inflammatory skin diseases or wound healing could result in a pH of less than 5.0 [11]. Thus, the improvement of the fluorescent materials could be necessary to expand the dynamic range of the glucose measurement at low pH conditions. 

To improve accuracy, a remaining issue is to increase the uniformity in the size and shape proportions among the compartments of the microbeads. The fluorescent signal intensity increases, and the response speed decreases with the increase in microbead diameter. On the other hand, the fluorescent signal intensity decreases, and the response speed increases with the decrease in microbead diameter. Considering these influences, hydrogel glucose sensors in the scale of several hundred micrometers [8,9] are appropriate for implantable transdermal fluorescent sensors. In addition, our fabricated microbeads were not symmetrical (Figure 3B) because of the difference in the viscosities of the two pre-gel solutions, which may have affected the accuracy of the fluorescence intensity measurements. We expect that narrower diameter distribution minimizes the influence on fluorescence intensity by improving the accuracy of glucose sensing. This problem could be solved by adjusting the physical characteristics of pre-gel solutions for both the pH sensor and the glucose sensor. Furthermore, we also expect that the addition of a reference fluorescent compartment to the Janus particle could enable us to increase the accuracy. For instance, three-compartment microbeads [13], including a glucose sensor, a pH sensor and a reference compartment that is not affected by any surrounding factors, can achieve more precise and stable glucose monitoring.

Besides the boronic acid-based fluorescent glucose-sensing polymer that we used in this work, other types of fluorescent glucose sensors have been developed recently: fluorescent glucose sensors based on glucose oxidase [20] or glucose binding protein (GBP) [21] use specific enzymes/GBPs during the testing process. Those types of sensors have a higher specificity to glucose than that of fluorescent glucose sensors that do not use proteins. On the other hand, specific pathological conditions and the degradation of the protein would influence long-term sensor performance. In addition, fluorescent measurement with in situ pH calibration has yet to be developed. Our glucose sensor is expected to be biostable, long lasting [9] and fully implantable, with the simultaneous multi-sensing of glucose and pH to increase accuracy, showing the potential of being a good alternative to the existing glucose-sensing methods for CGM. In addition to those fluorescent-based sensing methods, recently developed glucose sensors targeting body fluids such as saliva and tear fluids are often integrated with a wearable device [22,23,24,25,26]. Applying our fluorescent hydrogel glucose-sensing microbeads to improved wearable devices for measuring such body fluids could also widen the possibilities of stable and patient-friendly CGMs.

## 5. Conclusions

We succeeded in fabricating glucose sensor microbeads with pH calibration by using a Janus structure. The diameter of the device was precisely controlled, and we found that the fluorescence intensity of the microbeads changed depending on the pH values and the glucose concentrations. Furthermore, the experimental results showed the accurate measurement of glucose concentration by these new glucose sensor microbeads. We believe that this device can provide a new route for continuous glucose monitoring with high accuracy, thus preventing diabetic complications and improving the quality of life for people with diabetes.
